# Alginate-Based Aerogel Particles as Drug Delivery Systems: Investigation of the Supercritical Adsorption and In Vitro Evaluations

**DOI:** 10.3390/ma13020329

**Published:** 2020-01-10

**Authors:** Daria Lovskaya, Natalia Menshutina

**Affiliations:** International Center for transfer of Pharmaceutical and Biotechnology, Mendeleev University of Chemical Technology of Russia, Moscow 125480, Russia; chemcom@muctr.ru

**Keywords:** aerogel, supercritical fluids, adsorption, drug delivery systems, amorphization, in vitro tests, X-ray analysis

## Abstract

The present work focuses on the preparation of alginate-based aerogels in the form of particles for their further study as potential drug delivery systems (solid dosage forms). The dripping method was used to prepare certain gel particles, and supercritical drying was used to obtain final alginate-based aerogel particles. Three model active substances (ketoprofen, nimesulide, loratadine) were impregnated into the obtained aerogels using the supercritical adsorption process. Using the method of X-ray analysis, it was shown that the in the obtained drug-loaded aerogels the corresponding active substances are in an amorphous state, and the stability of this state after six months of storage is confirmed. In vitro dissolution tests for obtained drug-loaded aerogels was performed. For each sample, an appropriate dissolution medium (with certain pH) was determined. In vitro investigations showed the increasing of the release rate for all model active substances. Time was required to release and dissolve 50% of the active drug from drug-loaded aerogels (T_1/2_), reduced in comparison with pure active drugs in crystalline form. Obtained results provide insight into the application of alginate-based aerogel particles as a drug delivery system to improve pharmacokinetic properties of certain active drugs.

## 1. Introduction

Aerogels are porous materials with high adsorption capacity, which makes it possible to successfully impregnate various substances into aerogels: Active pharmaceutical ingredients [1,2,3], oils [4], additives for the food industry, etc. [5,6]. Organic aerogels in the form of particles are of the greatest interest, since for many potential applications, this is the most attractive form. For example, organic aerogels in the form of particles can be used in the pharmaceutical industry as drug delivery systems, in medicine as medical products (surgical sorbents), and in the food industry as a carrier for vitamin supplements or flavors.

Various polysaccharides (cellulose, starch, alginates, pectin) [7,8,9], as well as various proteins (egg, whey protein, etc.) [10] can be used to prepare organic aerogels. All these substances are united by the ability to form gels either in the presence of water and/or a crosslinking agent, or under the influence of temperature and other factors. After removing the liquid (supercritical drying process), dried solid particles of organic aerogels of a given size are obtained. One of the most promising materials for the preparation of aerogels that can be used as drug delivery systems is sodium alginate. Sodium alginate is a biocompatible and biodegradable polysaccharide, which is used in medicine, as well as a food additive. A number of studies have confirmed the safety of sodium alginate for the human, which makes it possible to use it in various industries.

The possibility of obtaining aerogels based on various organic substances, such as alginate, chitosan, various proteins, opens up the possibility of creating innovative materials with desired characteristics and possessing a number of important properties. These properties include high specific surface area, high porosity along with low density, high sorption capacity and, most importantly, biocompatibility, which guarantees complete safety for humans [11]. Of particular interest is the use of organic aerogels in the form of particles as drug delivery systems, since the impregnation of various active substances in aerogels allows obtaining compositions with improved pharmacokinetic properties, for example, with improved bioavailability, fast or, conversely, slow release of the active substance from the pores of the aerogel [12,13,14].

The main stage of the process of obtaining organic aerogels in the form of particles is the preparation of the mother solution followed by gelling with the help of a crosslinking agent [3,15,16,17]. Gelation can be caused by both chemical factors and physical (temperature, pH of the medium). After the formation of the gel, it is necessary to exchange the solvent, which is inside the porous structure of the gel, with the corresponding solvent, which must be dissolved in the medium of supercritical fluid. As a rule, these are various alcohols (ethyl, isopropyl) [1,3]. The final step is drying in a supercritical fluid (in most cases, it is carbon dioxide).

There are two main methods for obtaining particles of organic aerogels: Emulsion–gelation and dripping methods. The emulsion–gelation method is one of the most common methods for producing particles of aerogels [18,19]. However, one of the major drawbacks of the emulsion–gelation method for producing aerogel particles is the presence of an additional stage—separation from the oil. As a rule, multiple centrifugation is carried out for this purpose, which increases both the process time and energy consumption [19]. This is especially important when scaling from laboratory to semi-industrial or industrial levels. The dripping method consists in introducing a mother solution dropwise into a solution containing a crosslinking agent [20,21]. After dropping the solution, the process of gelation begins. After the formation of gel particles, it is necessary to keep the formed particles in solution with a crosslinking agent for 24 h in order for all chemical reactions to complete and no unreacted groups remain. Then, multistep solvent exchange of the initial solvent with the corresponding alcohol is carried out. The final stage is the process of supercritical drying. The advantages of this method include the absence of additional stages, such as separation from oil, as was shown for the emulsion–gelation method.

To obtain compositions based on aerogel with impregnated active substances, two main approaches can be distinguished: Impregnation before supercritical drying and impregnation into an already prepared aerogel, after the process of supercritical drying [3]. Of greatest interest is the second approach—supercritical adsorption. This method can be used only for those active substances that dissolve in the medium of the used supercritical fluid. The dissolved active substance diffuses into the pores of the aerogel and adsorbs on their surface [22,23,24]. The use of supercritical fluid technologies for the impregnation of active substances has many advantages [25]. The impregnation of active substances into the aerogel by supercritical adsorption is one of the most promising methods to improve such properties of pharmaceutical substances as bioavailability or the release time of the active substance [26]. Materials produced by supercritical adsorption can be used in the pharmaceutical, biomedicine, cosmetic and food industries, since the final product does not contain hazardous solvents. In addition, the use of supercritical fluids for the impregnation of active pharmaceutical ingredients, allows to obtain compositions with controlled drug delivery [27,28]. This allows to create a drug with a reduced content of the active substance (compared to already existing drugs), while the therapeutic effect after the use of this tool increases, as well as increases the safety for the patient [29,30].

Most of the active pharmaceutical ingredients are in a crystalline state, while the supercritical adsorption of substances into the pores of an aerogel may be a way to adsorb a substance mainly in the amorphous state. This is important from the point of view of improving the pharmacokinetic properties of the finished pharmaceutical composition, since when a substance is dissolved from an amorphous state, energy and time are not expended to destroy the crystal lattice. Compounds in the amorphous state are more chemically active, since they have large values of Gibbs energy due to the greater mobility of the molecules. According to the literature [11], when aerogel is destroyed in the body, the active substances adsorbed in the pores are released at a rate up to ten times greater than during normal dissolution from the solid dosage form. To determine the presence in the mixture of certain compounds (phases), it is necessary to conduct a qualitative X-ray analysis, which consists in analyzing the diffraction pattern given by the sample under study. The literature shows that the active pharmaceutical ingredients adsorbed into the pores of the aerogel do not give characteristic peaks of crystalline phases. Comparative X-ray patterns for crystalline ketoprofen, its mixtures with starch-based aerogel and ketoprofen adsorbed into pores of starch-based aerogel are given in [11]. It was shown that the peaks characteristic of the ketoprofen crystal sample are absent in the ketoprofen sample adsorbed on starch-based aerogel. Thus, it was concluded that ketoprofen in the aerogel is in an amorphous state. The loading of nifedipine (a hypotensive drug) into a pectin-based polysaccharide aerogel similarly affected the amorphization of the active substance [31]. Nifedipine in its crystalline form is poorly soluble in water and has a low bioavailability. In the amorphous state, this substance shows a high release rate and high bioavailability. In work [32], it was shown that fat-soluble vitamin D3 (cholecalciferol) adsorbed in sodium alginate-based aerogel is found in the pores of the aerogel in an amorphous state.

The possibility of using organic aerogels as drug delivery systems was first proposed in 1995 [33], and since then, interest has increased many times in the field of research of these materials and their use in the pharmaceutical industry [34,35,36]. The authors of [11] described a method for producing aerogels in the form of starch, alginate, and pectin-based microspheres and studying them as carriers of slightly soluble in water active substances for oral administration. The authors of [37] described a study on loading ketoprofen into a whey protein aerogel. Protein aerogels obtained during this study showed high mechanical strength, as well as good adsorption capacity. The results of the release behavior of the active substance from the aerogel showed a stable release of 60% ketoprofen at pH = 6.8. Depending on the aerogel used, it is possible to create drugs with both fast and slow release [3,38]. A variety of precursors for creating aerogels in combination with safety and nontoxicity makes aerogels very attractive materials for modern pharmaceuticals [28,39,40]. All these results are the starting point for the creation of new drug delivery systems with desired and controlled properties. For a single substance, you can choose the best aerogel with the necessary characteristics and get a modern, most effective, and completely safe drug that meets all the requirements of modern pharmaceuticals. Based on the review, it was concluded that aerogels are promising for use as drug delivery systems and studies on this topic are relevant.

## 2. Materials and Methods

### 2.1. Synthesis of Alginate-Based Gel Particles

To obtain gel particles, the dripping method was used as described in [41]. To prepare an aqueous solution of sodium alginate, food-grade sodium alginate (food supplement E401) was used. Calcium chloride (Sigma-Aldrich) was used as the source of calcium for the formation of a gel via the dripping method. At the first step, a certain amount of sodium alginate salt was mixed with distilled water to obtain the concentration 1 wt%. At this step the magnetic stirrer was used. Mixing was left overnight to ensure complete dissolution. At the second step, calcium chloride was mixed with distilled water using a magnetic stirrer to obtain the desired concentration (1 wt%). During the formation of gel particles, a solution of sodium alginate was introduced dropwise into a solution of calcium chloride through a needle under constant stirring. For the sake of completeness of the chemical reactions, the obtained gel particles were left in the solution of calcium chloride for 24 h. Multistep solvent exchange was carried out to prepare the gel particles for further supercritical drying. Isopropyl alcohol was used as an appropriate organic solvent and further alcohol concentration steps were used: 30, 50, 70, 90, 100, 100 wt%.

### 2.2. Supercritical Drying Procedure

**Figure 1:**
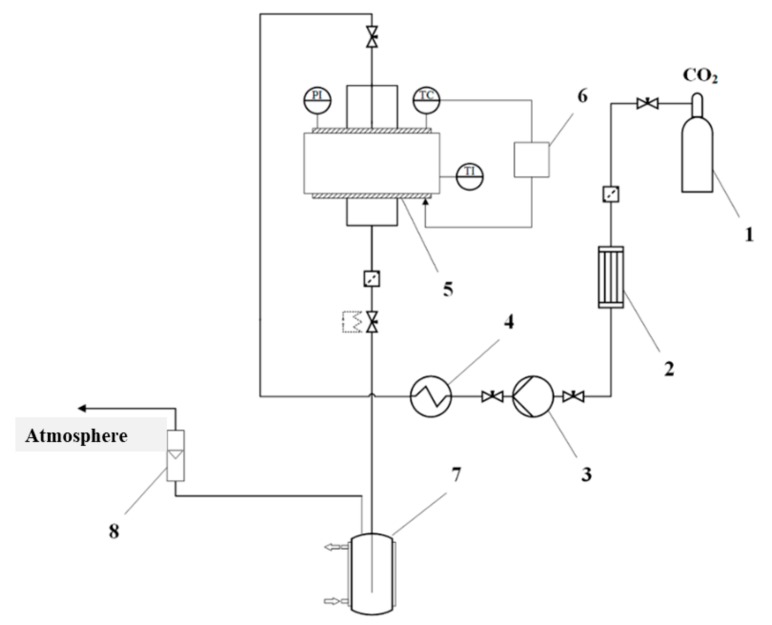
Scheme of the supercritical reactor. 1—CO2 tank, 2—condenser, 3—piston pump, 4—heater, 5—high pressure reactor, 6—thermal control system, 7—separator, 8—rotameter, PI—manometer, TC—temperature probe, TI—temperature probe.

Supercritical drying of gel particles was carried out in a high-pressure reactor with a volume of 0.25 L (own design). The scheme of the supercritical reactor is presented (Figure 1).

Process conditions: Temperature 40 °C, pressure 120–140 atm, flow rate 100 N L/h. Process time: 4–6 h.

### 2.3. Analytical Experiments

To determine the particle size distribution, an Analysette 28 ImageSizer (FRITSCH GmbH, Idar-Oberstein, Germany) was used. The average particle diameter was determined from the obtained differential curves of the particle size distribution. The textural characterization of the aerogels was carried out by low-temperature N2 adsorption—desorption analysis (ASAP 2020MP, Micromeritics, Norcross, GA, USA). Prior to measurements, samples were dried under a vacuum (<1 MPa) at 60 °C for 20 h. Specific surface area was determined by the BET (Brunauer-Emmett-Teller) method. BJH (Barrett-Joyner-Halenda) analysis was employed to determine average pore diameter using desorption techniques. Aerogel shape and appearance were analyzed using scanning electron microscopy (SEM) (JEOL 1610LV, (JEOL Ltd, Tokyo, Japan). Samples were platinum-sputtered (10 nm thickness) prior to imaging in order to minimize charging and improve the image contrast. Analytical experiments were performed at the core facilities centre of Mendeleyev University of Chemical Technology of Russia.

### 2.4. Adsorption Experiments

During this work, a number of adsorption experiments were performed with three model substances which are poorly soluble in water. They are ketoprofen, nimesulide, loratadine. 

**Figure 2:**
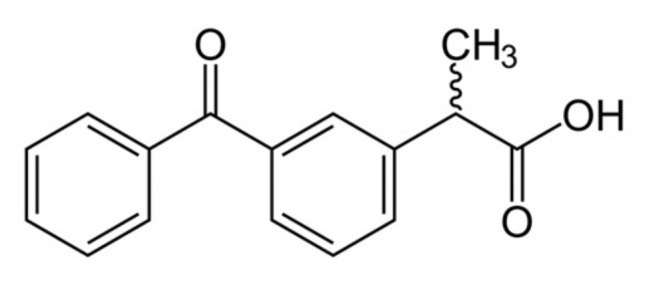
Ketoprofen structure.

Ketoprofen is a nonsteroidal anti-inflammatory drug (NSAID) from the group of propionic acid derivatives, which has an analgesic and antipyretic effect. The structure of ketoprofen molecule is shown (Figure 2).

**Figure 3:**
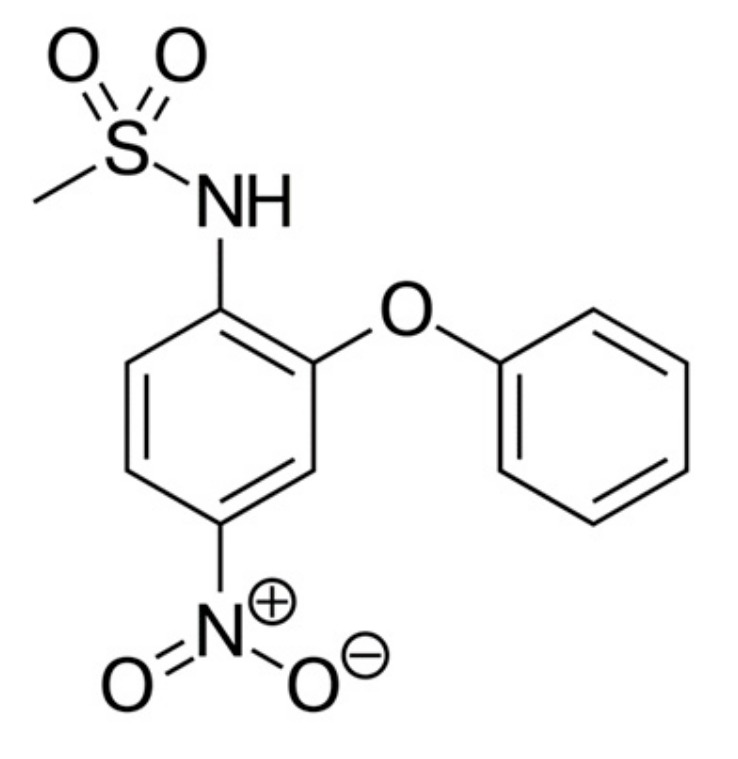
Nimesulide structure.

Nimesulide is the NSAID, has anti-inflammatory, analgesic, and antipyretic effects. The structure of the nimesulide molecule is shown (Figure 3).

**Figure 4:**
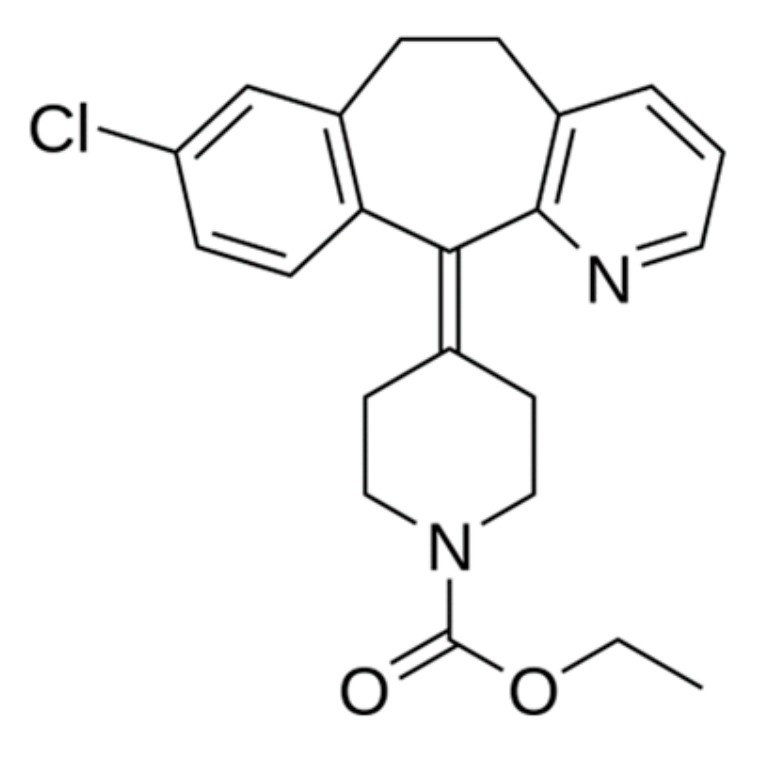
Loratadine structure.

Loratadine—antiallergic, antihistamine drug, is included in the list of essential and essential drugs. The structure of the molecule of loratadine is shown (Figure 4).

Table 1 provides reference data on the solubility of ketoprofen and nimesulide in supercritical carbon dioxide [42,43]. Data on the solubility of loratadine in supercritical carbon dioxide in the reference data are missing. However, an experimental study of the kinetics of supercritical adsorption of loratadine into particles of silica aerogel, which showed a high amount of adsorbed loratadine into aerogel, was carried out in [44].

**Figure 5:**
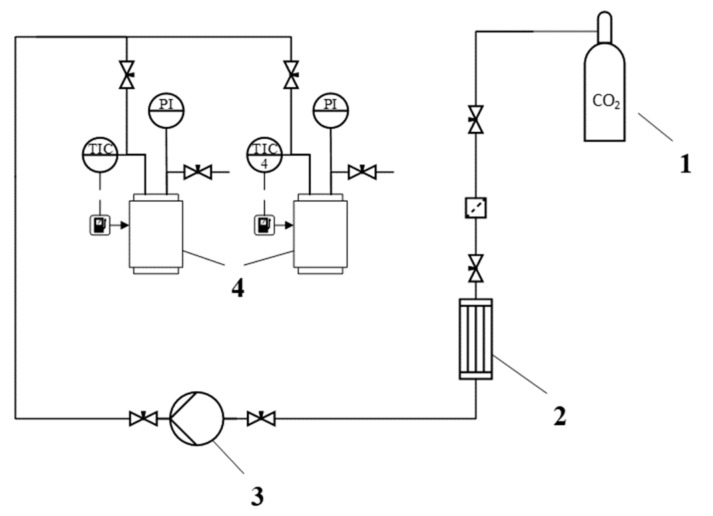
Scheme of the installation for supercritical adsorption: 1—CO2 tank, 2—heat exchanger, 3—liquid diaphragm pump, 4—high pressure reactors with heating jacket, PI—manometers, TIC—temperature controller with operator panel.

The adsorption process of active drugs was conducted in the supercritical high-pressure reactor (own design), the scheme of the installation is presented in Figure 5. The process was carried out as follows: Preweighed aerogel particles and a certain amount of API were placed in the two separate envelopes of filtering paper and loaded into the reactor. The mass of the API was calculated so that its amount is in excess in order to achieve the maximum adsorption value and create inside the apparatus an appropriate equilibrium concentration (solubility) in the entire range of the studied parameters. The apparatus was heated to a predetermined temperature and a slow current of liquefied carbon dioxide was fed to it. After reaching the predetermined pressure, the reactor was maintained for a specified amount of time. The process time was chosen on the basis of literature data on the adsorption of various active substances into alginate-based aerogel [8,40,45]. In all these works, it was shown that 48 h is sufficient to achieve adsorption equilibrium. This is the time of the process chosen in this experimental study.

The amount of adsorbed substance was determined by high performance liquid chromatography (HPLC). The analysis was performed on Agilent 1200 Compact LC C-18 column (4.5 × 12 mm, 5 μm). The mobile phase consisted of water, acetonitrile, and orthophosphoric acid (ratio 660:340:0.5); flow rate 2.0 mL/min; column temperature 40 °C. A detailed technique is given in the work [29].

### 2.5. X-ray Analysis

In the framework of this work, a series of experiments was carried out in the X-ray diffraction laboratory of the A. N Nesmeyanov Institute of Organoelement Compounds of Russian Academy of Sciences (INEOS RAS). The survey was carried out on a Bruker D8 Advance X-ray powder diffractometer (Bruker Corporation, Billerica, MA, USA), in the Bragg-Brentano geometry with variable mechanized slots, a nickel filter, and a LynxEye position-sensitive detector (Bruker Corporation, Billerica, MA, USA). Scanning was performed in a wide angular range in order to identify the largest number of diffraction maxima. The diffraction curve of the sample (diffractogram) is the dependence of the intensity of the diffracted X-ray beam on the double Bragg angle 2θ. When conducting X-ray phase analysis in the framework of this work, the following conditions were met: Wavelength 1.5418 Å (Cu Kα); shooting step 0.02° 2θ; the survey was carried out in the angular range of 4–65° 2θ with sample rotation.

### 2.6. In Vitro Dissolution Test

In vitro dissolution test was performed using Sotax AT7 Smart Dissolution Tester (Allschwil, Switzerland). The testing time for each sample was 60 min (following the recommendation of RUS Pharmacopeia). Previously, for each sample, an appropriate dissolution medium (with certain pH) was determined. 

According to FDA for the dissolution of aerogels loaded with ketoprofen and nimesulide phosphate buffer was used (pH = 6.8). Composition of phosphate buffer: Monobasic potassium phosphate, sodium hydroxide, deionized water. For the dissolution of aerogels loaded with loratadine (according to FDA) the model of gastric liquid was used (pH = 1.2). Composition of gastric liquid: Hydrochloric acid, sodium chloride, deionized water. The volume of the dissolution medium was 900 mL, the temperature was 37 °C. The rotation speed was 100 rpm. Sampling time—every 5 min after the start of the test. The sample volume was 5 mL. Quantitative determination was carried out using spectral analysis on a spectrophotometer ThermoScientific Evolution 300 UV–Vis. Previously, the wavelengths that corresponds to the maximum of the absorption spectrum of certain drug were determined. For ketoprofen, the maximum optical density of the peak corresponds to a wavelength of 298 nm, for nimesulide—389 nm, for loratadine—271 nm.

The dependence «concentration of released drug vs. time» was determined for certain drug-loaded aerogel. Moreover, it was determined that time was required to release 50% of the active drug from aerogel. The obtained data was compared with the same data for certain drug in crystalline form. After that was calculated, a reduction of time was required to release 50% of the active drug.

## 3. Results and Discussion

### 3.1. Results of Analytical Experiments

**Figure 6:**
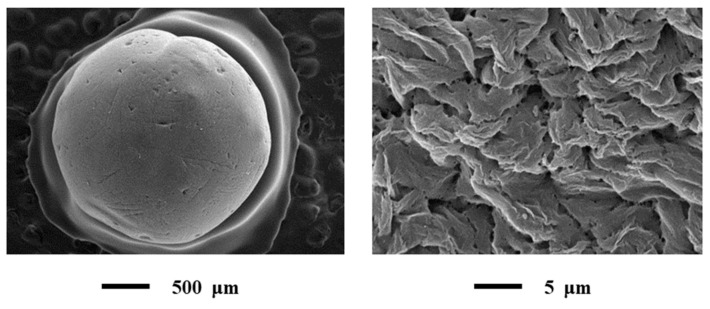
SEM of alginate-based aerogel particles: Single particle (on the left) and outer surface of the particle (on the right).

The results of SEM presented in Figure 6.

All SEM images acquired at low and high magnifications. 

Characteristics of the obtained aerogels are presented in Table 2. Data were obtained according to the procedure described in Section 2.3. 

The obtained results revealed that obtained alginate-based aerogel particles have a high specific surface area which allows using these particles as carriers for active drugs.

### 3.2. Results and Discussion of Adsorption Experiments

During the experimental study of the adsorption process of model active substances, the external parameters of the process varied: Temperature and pressure. The process temperature was chosen so that, on the one hand, carbon dioxide was in a supercritical state, and on the other hand, to prevent possible thermal decomposition of the active substances. Therefore, the process was carried out at temperatures of 40 and 60 °C. The process pressure was chosen within wide limits and amounted to 120 and 200 atm. The results of the experimental study are presented in Table 3.

#### 3.2.1. Ketoprofen Adsorption into the Alginate-Based Aerogel Particles

The results of supercritical adsorption showed the dependence of the amount of ketoprofen in the alginate-based aerogel on pressure: The higher the pressure, the higher the loading of the active substance into the aerogel. The loading increases by an average of 1.5 times. In addition, the results show that the loading increases slightly with increasing temperature. 

One of the factors that can affect the loading of the active substance into the aerogel is the solubility of this substance in supercritical carbon dioxide. If the solubility of ketoprofen is considered with the parameters of an experimental study, it can be seen that with increasing pressure, solubility increases on average by 4.6 times at different temperatures. With an increase in temperature, the solubility changes less significantly: At a pressure of 120 atm, the change can be neglected, and at a pressure of 200 atm, the solubility increases 1.6 times. It is likely that such a less significant change in solubility with temperature can explain the insignificant effect of temperature on the loading of ketoprofen into the aerogel. Conversely, the significant effect of pressure on the resulting amount of active substance can be explained by a significant change in solubility.

Another factor that can influence the amount of active substance in the aerogel is the presence (or absence) of interaction between the surface of the corresponding aerogel and the surface of the corresponding active substance. On the surface of the alginate-based aerogel are hydroxyl groups, and in the ketoprofen molecule there are carboxyl and carbonyl groups. Carboxyl group combines two functional groups—carbonyl and hydroxyl, mutually affecting each other. The electronic structure of the –COOH group gives carboxylic acids characteristic chemical and physical properties. The shift of the electron density to the carbonyl oxygen atom causes an additional polarization of the O-H bond, which determines the mobility of the hydrogen atom (this gives acidic properties). Hydrogen and oxygen atoms in the carboxyl group –COOH are capable of forming intermolecular hydrogen bonds, which largely determines the physical properties of carboxylic acids. Thus, it can be assumed that the interaction of these groups determines the interaction in general between the surface of the alginate-based aerogel and ketoprofen.

#### 3.2.2. Nimesulide Adsorption into the Alginate-Based Aerogel Particles

On the basis of the experimental data obtained, a tendency similar to that of ketoprofen can be noticed. With increasing pressure, the loading of nimesulide into the aerogel increases on average by 2.7 times, and with increasing temperature at various pressures only a slight change is observed. If the influence of the solubility of nimesulide is considered in supercritical carbon dioxide with the parameters of an experimental study, it can be seen that with increasing pressure, the solubility increases on average by seven times at different temperatures. With increasing temperature, the solubility changes less significantly: At a pressure of 120 atm, the solubility decreases by 1.8 times, and at a pressure of 200 atm, it increases by 1.4 times. It is likely that such a less significant change in solubility with a change in temperature can explain the insignificant effect of temperature on the loading of nimesulide into the aerogel. Conversely, the significant effect of pressure on the resulting amount of active substance can be explained by a significant change in solubility.

Next, we consider the possible interaction of groups in the nimesulide molecule with groups on the surface of the alginate-based aerogel. Nimesulide belongs to the class of sulfon-anilides. The hydrogen atom of the amino group in nimesulide is replaced by a methyl sulfonic acid residue. The interaction of this methylsulfonyl group in the composition of nimesulide with hydroxyl groups, which are present in the composition of the alginate, is insignificant. Thus, it can be assumed that the interaction of the entire nimesulide molecule with the surface of alginate-based aerogel in this case will be minimal. Probably due to the fact that the interaction measure in the aerogel-nimesulide pair is less than that in the aerogel-ketoprofen pair, the amount of active substance is also less.

If the results with the obtained loading of ketoprofen under different conditions are compared, then it is necessary to compare their solubility in supercritical carbon dioxide. On average, under various conditions, the solubility of nimesulide is 1.8 times lower than the solubility of ketoprofen. At the same time, the loading of nimesulide is lower than the loading of ketoprofen at a pressure of 120 atm by an average of 3.4 times, and at a pressure of 200 atm by 1.4 times.

Thus, if the results obtained for nimesulide and ketoprofen are compared, then it is impossible to make an unequivocal conclusion that it has the greatest influence on the difference in the resulting loadings. It is likely that the difference in solubility of these substances in supercritical carbon dioxide and the difference in the measures of their interaction with the surface of the alginate-based aerogel makes a significant contribution to the process of supercritical adsorption.

#### 3.2.3. Loratadine Adsorption into the Alginate-Based Aerogel Particles

With an increase in pressure, the amount of loratadine in the alginate-based aerogel increases on average by 1.2 times, and with a change in temperature at different pressures, a slight change in the loading is observed. Due to the lack of data on the solubility of loratadine in supercritical carbon dioxide, it is impossible to estimate the effect of a change in solubility with external parameters on a change in the loading.

The results obtained for aerogel compositions with loratadine showed a relatively high amount of active substance (the maximum loading 30.58 wt%). Loratadine has pronounced hydrophobic properties (due to the presence of nonpolar groups in its molecule). The hydroxyl groups on the surface of alginate-based aerogel can interact with the active groups of loratadine, which is a higher carboxylic acid ester. The carbon atom of the carbonyl group of loratadine is electrophilic, which may influence the interaction with active groups on the surface of aerogel. It can be assumed that the presence of the interaction in this case has a positive effect on the loading of loratadine into alginate-based aerogel particles.

Thus, for all model active substances that were used during the experimental study of the process of supercritical adsorption, a similar dependence on various factors is shown. In all cases, the loading increases with increasing pressure and varies slightly with temperature. However, this change may be due to a corresponding change in the solubility of the active substances. Despite the fact that the dependence of loading on solubility is qualitatively traced in all cases, it is difficult to quantify it. Therefore, the contribution of the solubility of the active substance to the course of the adsorption process is not completely clear compared to other factors: External process parameters (pressure and temperature), the magnitude of the interaction between the active substance, and the aerogel surface. Supercritical adsorption is a process of high complexity, the course of which depends on many factors, therefore, in the future, these studies will be continued.

### 3.3. Results and Discussion of X-ray Analysis

**Figure 7:**
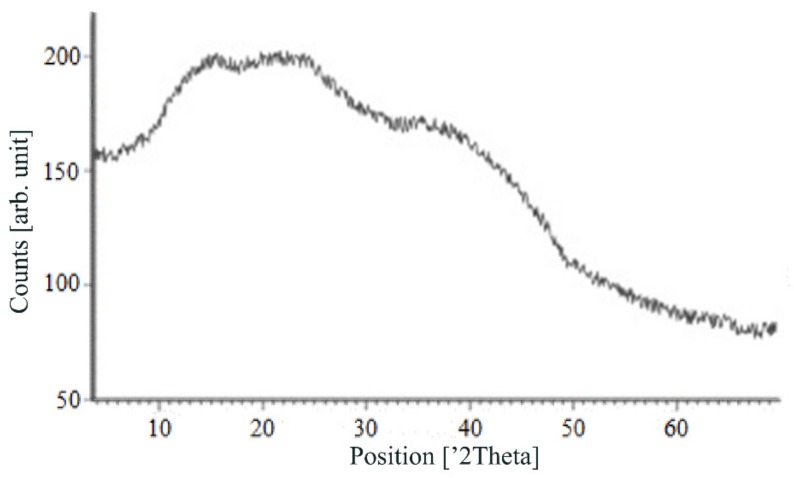
X-ray diffraction patterns of pristine alginate-based aerogel.

**Figure 8:**
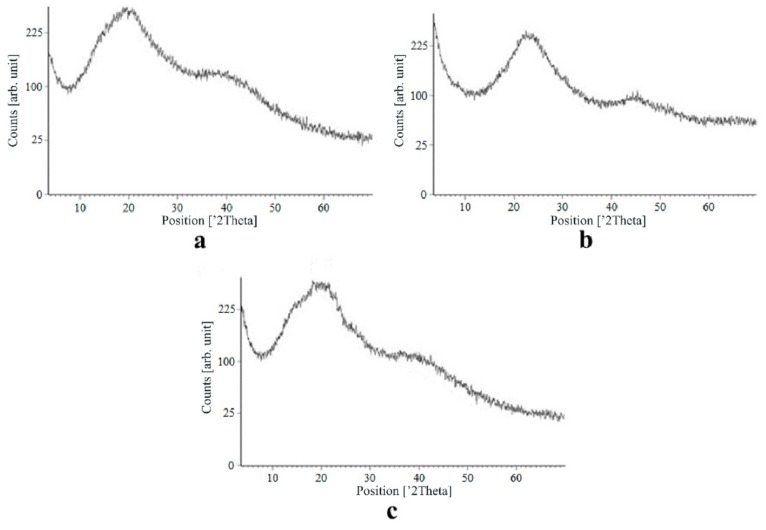
X-ray diffraction patterns of ketoprofen loaded into the alginate-based aerogel: (**a**) 18.05, (**b**) 19.36, (**c**) 26.98 wt%.

**Figure 9:**
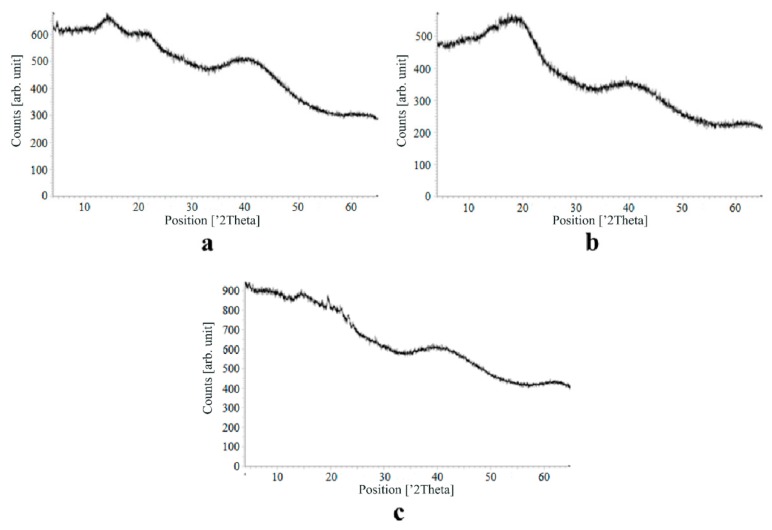
X-ray diffraction patterns of nimesulide loaded into the alginate-based aerogel: (**a**) 5.50, (**b**) 5.56, (**c**) 14.96 wt%.

**Figure 10:**
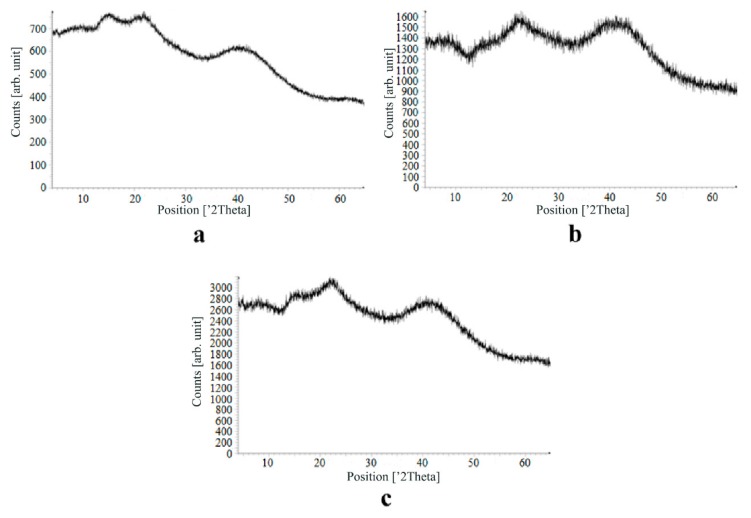
X-ray diffraction patterns of loratadine loaded into the alginate-based aerogel: (**a**) 24.32, (**b**) 26.92, (**c**) 30.55 wt %.

Figure 7, Figure 8, Figure 9 and Figure 10 show the results of X-ray analysis (diffraction patterns) for the previously obtained pristine alginate-based aerogel particles and drug-loaded alginate-based aerogel particles with different amounts of a certain active substance.

On X-ray diffraction patterns for all obtained drug-loaded alginate-based aerogel particles with different amounts of a certain active substance, there are no distinct peaks corresponding to the crystalline state of the substance. The resulting X-ray diffraction patterns can be seen as noise track, the occurrence of which is explained by the unevenness of the process of emission of quanta by X-ray tube or intrinsic noise of the detector. The final X-ray diffraction pattern for each sample was obtained by overlaying several diffraction patterns (for each sample, the experiment was repeated). The results of the qualitative X-ray analysis suggest that the drugs loaded into the alginate-based aerogel particles are mainly in the amorphous state. 

It can be seen from obtained data for all the samples obtained, both for the pristine aerogel and for drug-loaded aerogels, that there is a maximum region at 35°–45° 2Θ. Additionally, there are different diffraction patterns of drug-loaded aerogels at an angle less than 35° 2Θ. For ketoprofen-loaded aerogel, the location of the diffraction line is higher than for pristine aerogel in the range of angles from 12 to 25° 2Θ. In paper [11] characteristic peaks of crystalline ketoprofen are shown at 6,4° 2Θ in the range of angles from 12 to 30° 2Θ. This, probably, can explain the higher location of the diffraction line, for example, if few amounts of ketoprofen inside aerogel is in the form of nanocrystals. It is also interesting to note that in referenced work ketoprofen was adsorbed into starch-based aerogel and it was shown that diffraction patterns of pristine starch aerogel and ketoprofen loaded starch-based aerogels were practically identical. This difference may be due to different interactions of the ketoprofen molecule with starch and alginate molecules during supercritical adsorption and with different structural characteristics of these different aerogels. In referenced work starch-based aerogel has an average pore size of 14–18 nm, but in present work the alginate-based aerogel has an average pore size of 27 nm. To look through at the diffraction patterns of crystalline nimesulide [46,47], it can be seen that it has distinct peaks in the range from 5 to 20° 2Θ. Similarly, to ketoprofen, this can explain the higher location of the diffraction line in this region in comparison with pristine alginate-based aerogel. Probably, some amount of nimesulide may also be in the form of nanocrystals. The same situation is observed with loratadine, since its diffraction pattern has distinct peaks in approximately the same range of angles [48]. Thus, it cannot be argued that the drugs adsorbed into alginate-based aerogel are completely in an amorphous state, perhaps some of them are in the form of nanocrystals. However, even if nanocrystals of the drug take place, the release rate of such a drug should increase compared to the initial crystalline powder due to the significantly larger surface of the resulting dispersed system. It is important to note that obtained results allow only a qualitative analysis of drugs state adsorbed into aerogel and subsequent studies in this area are promising. 

The main disadvantage of the amorphous state of a substance is the instability of such a state. The crystalline state of matter is always more stable, whereas for amorphous states a spontaneous transition from an amorphous state to a crystalline state can be observed. This explains the need to test the drug-loaded compositions obtained for the stability of the amorphous state of a substance after a specified period of time. During the storage of drug-loaded aerogel particles, all of the above factors can affect these samples. In order to assess the state of the active substance in obtained drug-loaded alginate-based aerogel particles, a series of repeated studies were carried out using X-ray analysis. The resulting samples were stored for six months at room temperature. In addition, the analyzed samples were subjected to mechanical stress, which, as a rule, is accompanied by the process of transportation and storage (shaking samples, being under the influence of solar radiation, temperature drops). On the obtained X-ray diffraction patterns for all drug-loaded aerogels obtained within the framework of present investigation, there are no peaks corresponding to the crystalline state of the substance after six months of storage.

The results of the qualitative X-ray analysis suggest that the model active substances loaded into the alginate-based aerogel particles mainly in the amorphous state after six months of storage, including after exposure to various external factors. Summarizing, it can be concluded that the storage of drug-loaded alginate-based aerogel particles for six months when exposed to various external factors did not affect the stability of the amorphous state of certain active substances.

### 3.4. Results of In Vitro Dissolution Test

**Figure 11:**
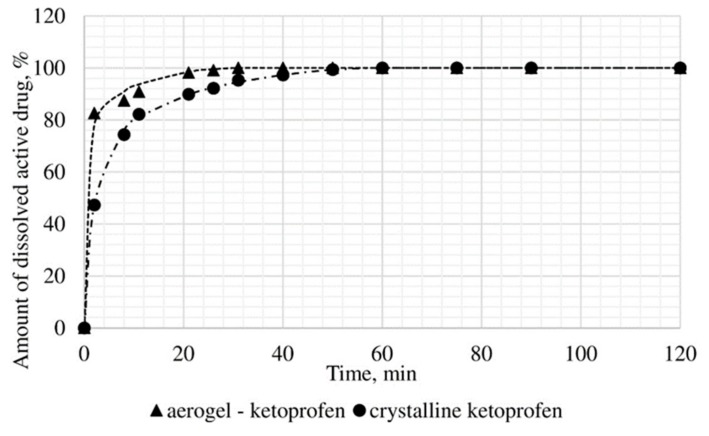
Results of in vitro dissolution test for ketoprofen loaded alginate-based aerogel.

**Figure 12:**
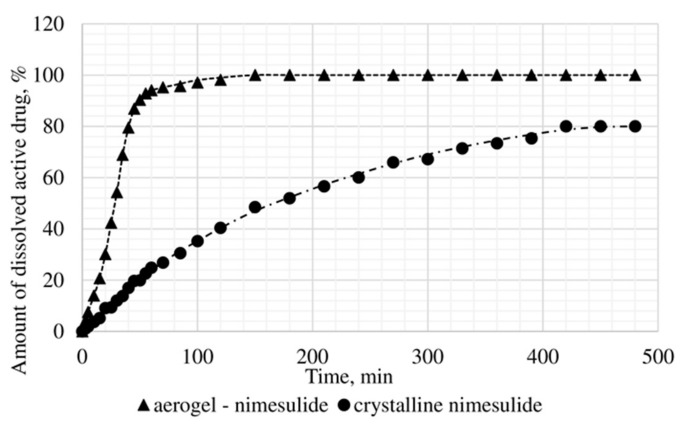
Results of in vitro dissolution test for nimesulide loaded alginate-based aerogel.

**Figure 13:**
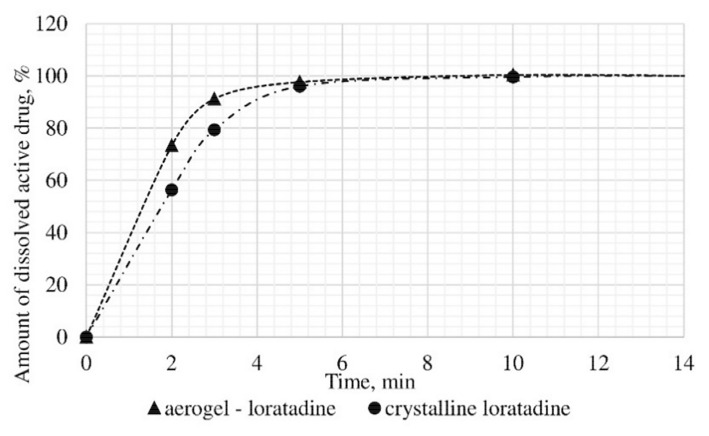
Results of in vitro dissolution test for loratadine loaded alginate-based aerogel.

The dissolution test was conducted, within which the kinetics of release of the certain active substance in the obtained drug-loaded aerogel, where the active substance is in the amorphous state, was studied, and compared with the dissolution kinetics of the corresponding active substances in the crystalline state. In vitro, the dissolution test was made into three samples: Ketoprofen loaded alginate-based aerogel (26.98 wt%), nimesulide loaded alginate-based aerogel (14.53 wt%), loratadine loaded alginate-based aerogel (26.92 wt%). Figure 11, Figure 12 and Figure 13 presents model active substances release from alginate-based aerogel particles. Lines in figures are an approximation of the corresponding experimental data.

A reduction of time for all investigated drug-loaded aerogels was measured. This time is required to release and dissolve 50% of model active substance (T_1/2_). When compared to T_1/2_ for drug loaded into aerogel with T_1/2_ for pure drug (Table 4), it could be observed that release of the aerogel-loaded drug is faster, and that this drug goes faster into the solution.

On the basis of the results of the study of the release kinetics for drug-loaded aerogels, it can be concluded that the active substances impregnated into the aerogel pass into the model solution much faster than their crystalline analogs. Comparison of the release profiles of compositions with the release profiles of similar pure substances showed a reduction in the release time of 50% (T_1/2_) of the active substance up to 6.6 times. This suggests that the supercritical adsorption of active substances into the aerogels makes it possible to obtain pharmaceutical forms of drugs mainly in an amorphous state with improved pharmacokinetic properties. Obtained results showed that supercritical adsorption of active pharmaceutical ingredients into the alginate-based aerogel particles could improve the dissolution rate, accelerating the release time and providing a faster therapeutic effect. These effects are especially important for studied drugs, since they are poorly soluble in water. Alginate-based aerogel particles as carriers for active pharmaceutical ingredients have a positive effect on the pharmacokinetic properties of the drug substance.

## 4. Conclusions

A study of the preparation of alginate-based aerogels in the form of particles using the dripping method with subsequent supercritical drying procedure was conducted. Obtained aerogel samples were investigated using various analytical methods; the value of specific surface area which was 512 m^2^/g was determined. 

Three model active substances (ketoprofen, nimesulide, loratadine) were impregnated into the obtained aerogels using the supercritical adsorption process. The amount of active drugs adsorbed into aerogels were determined by the HPLC method.

During the experimental study of the adsorption process of model active substances, the external parameters of the process varied: Temperature (40 and 60 °C) and pressure (120 and 200 atm). In all cases, the loading increases with increasing pressure and varies slightly with temperature. Considered is the possible impact of the presence (or absence) of interaction between the surface of the aerogel and the surface of the corresponding active substance on the amount of active substance in the aerogel.

The results of the qualitative X-ray analysis suggest that the drugs loaded into the alginate-based aerogel particles are mainly in the amorphous state. However, presence probability of nanocrystals was shown, since diffraction patterns of drug loaded alginate-based aerogels have a difference in comparison with pristine aerogel. The storage of drug-loaded alginate-based aerogel particles for six months when exposed to various external factors did not affect the stability of the amorphous state of certain active substances.

In vitro dissolution tests were performed for the obtained drug-loaded aerogels. The dependence «concentration of released drug vs time» was determined. Obtained results showed that release rate increased for ketoprofen, nimesulide, and loratadine loaded aerogels, respectively. Comparison of the release profiles of compositions with the release profiles of similar pure substances showed a reduction in the release time of 50% (T_1/2_) of the active substance up to 6.6 times. This suggests that the supercritical adsorption of active substances into the aerogels makes it possible to obtain pharmaceutical forms of drugs in a stable amorphous state with improved pharmacokinetic properties.

Based on the obtained results it could be concluded that alginate-based aerogel particles can be promising for the pharmaceutical industry as potential drug delivery systems.

## Figures and Tables

**Table 1:** Solubility data of selected API (ketoprofen and nimesulide) in supercritical carbon dioxide (y—mole fraction solubility (mole solute/mole mixture)).

API	Parameters		Solubility, y × 10^6^
	Temperature, °C	Pressure, atm	
Ketoprofen [43]	40	110	21.2
		130	55.2
		200	87.8
		250	91.5
	55	150	46.6
		170	70.3
		190	103.0
		230	161.0
		250	188.0
Nimesulide [44]	40	130	18.9
		160	31.8
		190	51.1
		220	74.2
	60	130	08.5
		160	38.0
		190	70.8
		220	98.5

**Table 2:** Characteristics of the obtained alginate-based aerogel particles.

Average Particle Diameter, μm	Specific Surface Area, m^2^/g	Average Pore Diameter, nm
2700 ± 100	512 ± 2.15	27

**Table 3:** Results of adsorption experiments.

Active Substance	Temperature, °C	Pressure, atm	Loading, wt %
Ketoprofen	40	120	18.05
	40	200	26.98
	60	120	19.36
	60	200	28.95
Nimesulide	40	120	5.56
	40	200	14.53
	60	120	5.50
	60	200	14.96
Loratadine	40	120	24.32
	40	200	30.58
	60	120	26.92
	60	200	30.55

**Table 4:** Results of in vitro dissolution test.

Drug-Loaded Aerogel	T_1/2_, min	Reduction of T_1/2_
Ketoprofen loaded alginate-based aerogel	1.2	1.6
Crystalline ketoprofen	1.9	
Nimesulide loaded alginate-based aerogel	25	6.6
Crystalline nimesulide	165	
Loratadine loaded alginate-based aerogel	0.5	2.2
Crystalline loratadine	1.1	
